# The efficacy of intravenous sodium valproate and phenytoin as the first-line treatment in status epilepticus: a comparison study

**DOI:** 10.1186/1471-2377-13-98

**Published:** 2013-07-27

**Authors:** Somsak Tiamkao, Kittisak Sawanyawisuth, Alongkorn Chancharoen

**Affiliations:** 1 Department of Medicine, Faculty of Medicine, Integrated Epilepsy Research Group, Khon Kaen University, Khon Kaen, Thailand; 2 Department of Medicine, Faculty of Medicine, Khon Kaen University, 123 Mitraparp Road, Khon Kaen 40002, Thailand

**Keywords:** Phenytoin, Sodium valproate, Efficacy, Status epilepticus, Comparison

## Abstract

**Background:**

Status epilepticus (SE) is a serious neurological condition and requires prompt treatment. Sodium valproate has been used to treat SE successfully but its role as the first-line antiepileptic drug (AED) is still controversial. This study evaluated the efficacy of intravenous sodium valproate to determine if it is non-inferior to intravenous phenytoin in SE treatment.

**Methods:**

Patients diagnosed as SE during 2003–2010 who were of an age of more than 15 years and received either intravenous sodium valproate or intravenous phenytoin as the first-line treatment were enrolled. Clinical characteristics and outcomes of SE were recorded and analyzed. The differences of outcomes between sodium valproate and phenytoin group were determined by descriptive statistics.

**Results:**

During the study period, there were 37 and 17 SE patients who received intravenous phenytoin and intravenous sodium valproate as the first-line treatment, respectively. All patients received diazepam 10 mg intravenously as a rescue medication before starting the antiepileptic agents if uncontrolled except one patient in the sodium valproate group. There were no significant differences between the phenytoin and sodium valproate groups in all outcome variables including numbers of patients with clinically-controlled seizures, non-dependent patients, time to seizure control, and duration of hospitalization, and death. No serious cardiovasculars event such as hypotension occurred in either group.

**Conclusion:**

Intravenous sodium valproate is non-inferior to intravenous phenytoin as the first-line treatment in SE with no significant cardiovascular compromises.

## Background

Status epilepticus (SE) is an emergency condition that requires proper and prompt treatment to prevent morbidity and mortality. Intravenous phenytoin is a main medication to treat SE. Recent and new antiepileptic drugs (AED) such as sodium valproate, lacosamide, levetiracetam or topiramate have potential benefits in treatment of SE [1-4].

Previous studies showed that intravenous sodium valproate may be a potential AED to be effective in SE [1,5]. It may be used as the first-line AED in SE with a good seizure control [5]. Unlike phenytoin [6-8], sodium valproate can be used safely and has no potential major cardiovascular compromises such as cardiac arrhythmia or hypotension [9-12]. Sodium valproate therefore may be an appropriate drug as the first-line treatment in SE.

A meta-analysis of five randomized-controlled studies showed that both intravenous phenytoin and sodium valproate were effective in SE treatment [1]. This study aims to investigate the efficacy of both medications in the treatment of SE as the first-line AED. The results will add more information of the efficacy of both drugs on SE in the literature. The study therefore evaluated if intravenous sodium valproate is non-inferior to intravenous phenytoin in SE treatment.

## Methods

SE patients treated at Srinagarind Hospital, Khon Kaen University, Thailand between 2003 and 2010 were enrolled. The inclusion criteria were patients with an age of more than 15 years and received either intravenous phenytoin or intravenous sodium valproate as the first-line treatment.

Status epilepticus (SE) is a condition with persistent seizure more than 5 minutes or the patient does not gain the consciousness during the interictal period [13]. Generalized convulsive SE is defined as recurrent convulsive seizures that may be overt or subtle, symmetric or asymmetric, and are associated with profound coma and bilateral, although often has asymmetric, ictal discharges on electroencephalogram (EEG) [14]. Non-convulsive SE is defined as SE with a change in behavior and/or mental processes from baseline associated or associated with continuous epileptiform discharges in the EEG or in response to treatment [15].

Data were retrieved from medical records including baseline characteristics, previous medical illnesses, causes of SE, laboratory findings, numbers of AED usage, and outcomes of treatment. The outcomes of treatment were numbers of patients with clinically-controlled seizure, time to seizure control, admission duration, patient status after treatment, change of functional status after treatment, and death. Time to seizure control was identified by the total minutes spent from the start of SE treatment to the time until there was no clinical evidence of seizure. Seizure control was defined clinically and/or by EEG. Patient status after treatment was defined by patient’s functional capacity at the discharge date from the hospital and categorized as dependent or non-dependent status. The dependent status was defined as a condition that the patients needed someone to assist them daily life activities and equal to modified Rankin scale (mRs) of two or more. Worsening of functional outcome was defined by change of functional outcome to dependent status or death at discharge date. Mortality rate and duration of treatment were recorded.

Baseline characteristics between phenytoin and sodium valproate group were compared by descriptive statistics. All outcome variables were also tested for the differences between those treated with phenytoin and sodium valproate. Wilcoxon rank sum or student *t*-test and Fisher’s exact tests or Chi-square test were applied to compare the differences in numbers and proportions between the two groups where appropriate. The study protocol was approved by the ethics committee on human research of Khon Kaen University (HE541319).

## Results

During the study period, there were 92 patients diagnosed as SE. Of those 37, 17 SE patients received intravenous phenytoin and 20 received intravenous sodium valproate as the first-line treatment. All patients received diazepam 10 mg intravenously as a rescue medication before starting antiepileptic agents if uncontrolled except one patient in the sodium valproate group.

There were no statistically significant differences in the baseline clinical variables between both groups (Table 1). The median age was higher and the median time to start SE treatment was longer in sodium valproate group. The percentages of patients with male gender, epilepsy, and antiepileptic drug withdrawal were also higher in sodium valproate group. EEG was done in 14 patients (25.93%). There were no statistically significant differences, however, in terms of causes and pre-existing conditions of SE in both groups (Table 2). Details of history of AED usage in patients with previous history of epilepsy are provided in Table 3.

**Table 1:** Baseline characteristics of status epilepticus (SE) patients treated with intravenous phenytoin or intravenous sodium valproate as the first-line treatment

**Variables**	**Phenytoin group N = 37**	**Sodium valproate N = 17**	**p value**
Age;(years)	40 (16–85)	42 (16–76)	0.852
Male gender (%)	17 (45.95)	10 (58.82)	0.379
No preexisting condition, N (%)	5 (13.51)	3 (17.65)	0.696
History of epilepsy (%)	6 (16.22)	7 (41.18)	0.084
Antiepileptic drug withdrawal (%)	4 (10.81)	5 (29.41)	0.121
Admitted to internal medicine ward (%)	14 (37.84)	6 (35.29)	1.000
Generalized tonic clonic seizure type (%)	37 (100)	16 (94.12)	0.315
Weight (kgs)*	55.5 (40–75)	50 (39–97.5)	0.343
Time to start treatment (minutes)	5 (5–90)	10 (5–35)	0.127
Independent status prior to SE, N (%)	28 (75.68)	17 (100)	0.177
Laboratory findings			
Hematocrit, g/dL*	31 (15.6-45.7)	36.5 (26–49.4)	0.273
Total white blood cells, cells/mm^3^*	11610 (4100–29700)	8965 (1100–14500)	0.168
Blood sugar, mg/dL*	127 (37–568)	131 (73–251)	0.926
Serum creatinine, md/dL*	1 (0.5-6.1)	0.9 (0.5-7)	0.445
Serum calcium, md/dL*	8.4 (5.3-10.2)	8.2 (0.9-10.7)	0.976
Serum albumin, g/dL*	3.3 (1.2-7.4)	4.0 (2.2-5.1)	0.072
ALT, U/L*	30 (5–375)	18 (4–165)	0.242
AST, U/L*	56 (11–473)	25.5 (0.3-2.2)	0.115
Creatinine kinase, U/L*	276 (54–1500)	174.5 (44–500)	0.396
Abnormal CT brain findings*	17 (58.62)	7 (53.85)	1.000
Electroencephalogram, N*	9 (24.32)	5 (29.42)	0.745

Note. Data presented as median (range) or number (percentage), Data in phenytoin or sodium valproate group may not equal to 37 or 17, respectively due to missing data, *ALT* serum alanine transaminase, *AST* serum aspartate transaminase, *CT* computed tomography, * indicates missing data.

**Table 2:** Causes of status epilepticus (SE) patients treated with intravenous phenytoin or intravenous sodium valproate as the first-line treatment

**Causes**	**Phenytoin group N = 37**	**Sodium valproate N = 17**	**p value**
Antiepileptic drug withdrawal	4 (10.81)	5 (29.41)	0.121
Sepsis	7 (18.92)	0	0.084
Uremia	0	1 (5.88)	0.315
Cardiac arrest	5 (13.51)	2 (11.76)	1.000
Alcohol withdrawal	1 (2.70)	0	1.000
CNS infection	9 (24.32)	3 (17.65)	0.584
Head injury	2 (5.41)	0	1.000
Metabolic causes	9 (24.32)	1 (5.88)	0.105
Others*	10 (27.03)	6 (35.29)	0.537

Note. Data presented as median (range) or number (percentage); *phenytoin group: hypoxic encephalopathy 3, CNS vasculitis 2, epidural hematoma 1, postcraniotomy 1, tuberculous encephalitis 1, venous thrombosis 1, ischemic stroke 1; sodium valproate group: postcraniotomy 2, saggital sinus thrombosis 1, subarachnoid hemorrhage 1, intracerebral hemorrhage 1, pregnancy with eclampsia.

**Table 3:** Antiepileptic drug usage in status epilepticus treated by intravenous phenytoin and intravenous sodium valproate as the first-line treatment

**Phenytoin group**	**Sodium valproate group**
**1. Phenytoin**	**1. Sodium valproate**
**2. Phenytoin, Phenobarbital**	**2. Sodium valproate**
**3. Phenytoin, Phenobarbital**	**3. Phenytoin, Phenobarbital**
**4. Phenytoin**	**4. Sodium valproate, Phenobarbital**
5. Phenytoin, Phenobarbital	**5. Sodium valproate, Phenytoin**
6. Phenytoin, Sodium valproate	6. Topiramate
	7. Topiramate

Note. Bold letter indicates status epilepticus from antiepileptic drug withdrawal.

The mean doses of AED used in phenytoin and sodium valproate group were 1.95 (SD 0.85) and 1.82 (SD1.01), (p value 0.508). The numbers of patients with two or more AED usage were 10 (27.02%) and 5 (29.41%) in the phenytoin and sodium valproate groups, respectively (Table 4). Details of AED order of usage in each group are shown in Table 5. The average loading dose of phenytoin was 743 (SD 116) mg with the infusion rate of 20.63 (SD 9.54) mg/min, while sodium valproate was given intravenously at average loading dose of 1000 (SD 239.14) mg and an infusion rate of 26.27 (SD 10.76) mg/min. Then, both medications were given intravenously with maintenance doses of 300 mg of phenytoin and 1,200 mg of sodium valproate. The serum phenytoin levels were measured in 16 patients. The average phenytoin level was 25.71 (SD 28.65) mg/dL, while the average sodium valproate level was 36.82 (SD 73.45) mg/dL (calculated from 7 patients).

**Table 4:** Numbers of antiepileptic drug (AED) used in status epilepticus patients by group

**No. AED**	**Phenytoin group N = 37**	**Sodium valproate group N = 17**
1	13 (35.14)	9 (52.94)
2	14 (37.84)	3 (17.65)
3	9 (24.32)	4 (23.53)
4	1 (2.70)	1 (5.88)

**Table 5:** Antiepileptic drugs used in status epilepticus patients by orders

**Order of antiepileptic drugs**	**Phenytoin group N = 37**	**Sodium valproate group N = 17**
Rescuer	Diazepam 37	Diazepam 16
First-line	Phenytoin 37	Sodium valproate 17
Second- line	Phenytoin 9	Sodium valproate 7
	Phenobarbital 8	Phenytoin 1
	Sodium valproate 7	
Third- line	Phenobarbital 7	Phenobarbital 5
	Sodium valproate 1	
	Sodium thiopenthal 1	
	Propofol 1	
Fourth- line	Phenobarbital 1	Sodium thiopenthal 1

Note. Numbers after each antiepileptic drug indicates number of patients.

Regarding the treatment outcomes (Table 6), there was no significant difference between the phenytoin and sodium valproate groups in all outcome variables. The differences of numbers of clinically-controlled seizure, however, was almost reached a statistical significant level (p value = 0.057). The time to seizure control, duration of hospitalization, the number of non-dependent patients or death were better in sodium valproate group (p value > 0.05) as shown in Table 6. There were 13 patients who died during the admission; 2 patients in the sodium valproate group. The median hospitalization days of all 13 patients were 9 days (range 3–31 days). Two patients died from acute renal failure and rhabdomyolysis, while the others died from septicemia. No serious cardiovascular eventd such as hypotension occurred in either group.

**Table 6:** Six outcome variables of status epilepticus patients treated by intravenous phenytoin or sodium valproate as the first-line treatment

**Outcomes**	**Phenytoin N = 37**	**Sodium valproate N = 17**	**p value**
Seizure controlled, N	8 (21.62)	8 (47.06)	0.057*
Time to seizure controlled (minutes)	30 (20–30)	20 (15–40)	0.173***
Hospitalization (days)	12 (3–109)	9 (2–76)	0.434***
Non-dependent status, N	20 (54.05)	13 (76.47)	0.143**
Worsening functional status, N	20 (54.05)	7 (41.18)	0.559**
Death, N	11 (29.73)	2 (11.76)	0.189**

Note. Data presented as median (range) or number (percentage); p value was calculated by *Chi-square, **Fisher Exact test, or ***Wilcoxon rank sum test.

## Discussion

This study showed that intravenous sodium valproate has non-inferior efficacy to intravenous phenytoin as the first-line treatment of SE. The intravenous sodium valproate group had better outcomes in all five variables (Table 6). Numbers of patients with clinically-controlled seizures was almost statistically significant (p value 0.057). A previous study by the present authors [5] showed that intravenous sodium valproate can control SE better if used as the first-line compared to the second-line treatment (75% vs 35%). Even though some causes or pre-existing conditions such as AED withdrawal induced SE may be easier to control [16], there was no statistical significance between the groups regarding these two factors (Tables 2 and 3).

The most different outcome between intravenous sodium valproate and phenytoin is the number of non-dependent status patients (22.42%). At baseline, patients in sodium valproate group were all independent, while 75.68% in phenytoin group were independent (p value 0.77). After having SE, the functional status at discharge of both groups were decreased (23.53% in sodium valproate group and 21.63% in phenytoin group, p value 0.143). The baseline functional status of patients in sodium valproate group was better phenytoin group (Table 1) but numbers of patients with worsening functional status (Table 6) was higher in phenytoin group (54.05% vs 41.18%, p value 0.559). The results of the number of patients with worsening of functional status at discharge suggested that patients in the sodium valproate group may have better functional outcomes at discharge.

None of the patients died during the seizure attack but the median survival was 9 days. Two patients died from SE related complications or rhabdomyolysis. Both patients were treated with phenytoin. The mortality rate in the sodium valproate group was much lower than phenytoin group (11.76% vs 29.73%; p value 0.189). Note that small numbers of patients in sodium valproate group. A further prospective study is therefore needed to confirm this finding. The non-significant results of all six outcomes are suggesting that sodium valproate was not inferior to phenytoin as the first-line treatment in SE as previously shown by the meta-analysis [1]. These findings are also compatible with a study from Iran with 30 SE patients. Both medications had comparable efficacy but phenytoin caused more non-serious or skin reactions at the injection site (26.7% vs 0%, p value 0.03) [17].

The reason that intravenous sodium valproate was used commonly in the KKU Hospital during the study period is due to a lack of intravenous phenobarbital. There were no serious cardiovascular compromises in patients who received intravenous sodium valproate. This indicated that intravenous sodium valproate is safe and effective to use as the first-line treatment in SE. Previous studies also showed comparable efficacy of intravenous sodium valproate in SE when compared to intravenous phenytoin [1,17].

There are some limitations to the present study. The retrospective study design had incomplete data collection. The numbers of patients in each group were also not comparable. Phynytoin is recommended as the first-line antiepileptic drug for status epilepticus in Thailand, while sodium valproate may be used in the elderly, patients with cardiovascular risks or hepatitis. Physicians therefore may choose phenytoin more often than sodium valproate. The outcomes, however, were better in the sodium valproate group. The other limitation is small number of patients in each group. In addition, a prospective study comparing intravenous phenytoin and intravenous sodium valproate as the first-line treatment in SE patients is needed to confirm that intravenous sodium valproate can be used as the first-line AED for recommendations in the SE guidelines.

## Conclusion

Intravenous sodium valproate is non-inferior to intravenous phenytoin as the first-line treatment in SE with no significant cardiovascular compromises.

## Competing interests

The authors declare that they have no competing interests.

## Authors’ contributions

ST: designed the study, verified data, interpreted data, and drafted the manuscript. KS: designed the study, analyzed data, and drafted the manuscript. AC: data collection. All authors read and approved the final manuscript.

## Pre-publication history

The pre-publication history for this paper can be accessed here:

http://www.biomedcentral.com/1471-2377/13/98/prepub
